# A review on slurry bioreactors for bioremediation of soils and sediments

**DOI:** 10.1186/1475-2859-7-5

**Published:** 2008-02-29

**Authors:** Ireri V Robles-González, Fabio Fava, Héctor M Poggi-Varaldo

**Affiliations:** 1 CINVESTAV-IPN, Environmental Biotechnology R&D Group, Dept. Biotechnology and Bioengineering, México D.F., México; 2 Alma Mater Studiorum-University of Bologna; Faculty of Engineering, Viale Risorgimento, 2. 40136. Bologna, Italy

## Abstract

The aim of this work is to present a critical review on slurry bioreactors (SB) and their application to bioremediation of soils and sediments polluted with recalcitrant and toxic compounds. The scope of the review encompasses the following subjects: (*i*) process fundamentals of SB and analysis of advantages and disadvantages; (*ii*) the most recent applications of SB to laboratory scale and commercial scale soil bioremediation, with a focus on pesticides, explosives, polynuclear aromatic hydrocarbons, and chlorinated organic pollutants; (*iii*) trends on the use of surfactants to improve availability of contaminants and supplementation with degradable carbon sources to enhance cometabolism of pollutants; (*iv*) recent findings on the utilization of electron acceptors other than oxygen; (*v*) bioaugmentation and advances made on characterization of microbial communities of SB; (*vi*) developments on ecotoxicity assays aimed at evaluating bioremediation efficiency of the process.

From this review it can be concluded that SB is an effective *ad situ *and *ex situ *technology that can be used for bioremediation of problematic sites, such as those characterized by soils with high contents of clay and organic matter, by pollutants that are recalcitrant, toxic, and display hysteretic behavior, or when bioremediation should be accomplished in short times under the pressure and monitoring of environmental agencies and regulators. SB technology allows for the convenient manipulation and control of several environmental parameters that could lead to enhanced and faster treatment of polluted soils: nutrient N, P and organic carbon source (biostimulation), inocula (bioaugmentation), increased availability of pollutants by use of surfactants or inducing biosurfactant production inside the SB, etc. An interesting emerging area is the use of SB with simultaneous electron acceptors, which has demonstrated its usefulness for the bioremediation of soils polluted with hydrocarbons and some organochlorinated compounds. Characterization studies of microbial communities of SB are still in the early stages, in spite of their significance for improving reactor operation and design optimization.

We have identified the following niches of research needs for SB in the near and mid term future, *inter alia*: (*i*) application of SB with sequential and simultaneous electron acceptors to soils polluted with contaminants other than hydrocarbons (*i.e*., pesticides, explosives, *etc.*), (*ii*) evaluation of the technical feasibility of triphasic SB that use innocuous solvents to help desorbing pollutants strongly attached to soils, and in turn, to enhance their biodegradation, (*iii*) gaining deeper insight of microbial communities present in SB with the intensified application of molecular biology tools such as PCR-DGGE, PCR-TGGE, ARDRA, etc., (*iv*) development of more representative ecotoxicological assays to better assess the effectiveness of a given bioremediation process.

## 1. Introduction

Bioremediation is an alternative that offers the possibility to destroy toxic pollutant using natural biological activity. By definition, bioremediation is the use of living organisms, primarily microorganisms, to degrade the environmental contaminants into less toxic forms. It uses naturally occurring bacteria and fungi or plants to degrade or detoxify substances hazardous to human health and/or the environment [1]. The microorganisms may be indigenous to a contaminated area or they may be isolated from elsewhere and brought to the contaminated site. Contaminant compounds are transformed by living organisms through reactions that take place as a part of their metabolic processes. Biodegradation of a compound is often a result of the actions of multiple organisms. When microorganisms are imported to a contaminated site to enhance degradation we have a process known as bioaugmentation [2]. Bioremediation techniques are typically more economical than thermal and physico-chemical remediation such as incineration [3,4].

Bioremediation processes have been classified in three broad categories, according to place and soil handling/conditioning: *in situ*, *ad situ*, and *ex situ*. The second and third class of processes are useful for the remediation of (1) sludges, soils or sediments polluted with high concentration of recalcitrant contaminants [5], for instance polynuclear aromatic hydrocarbons [6-9], diesel [10,11], explosives [12], pesticides and chlorinated organic pollutants [13,14], oily sludges from the petrochemical industry [15]; (2) clayish and stratified soils with low hydraulic conductivity and low permeability accompanied with high contents of organic matter [13,16]; and (3) soils in regions and areas where environmental conditions are adverse to biological processes, for instance, low temperature that negatively affects biodegradation rates [17], and (4) contaminated sites that require a short remediation time because of regulatory or other pressures [18-20].

Slurry bioreactors are one of the most important types of *ad situ *and *ex situ *technique. Treatment of soils and sediments in slurry bioreactors has become one of the best options for the bioremediation of soils polluted by recalcitrant pollutants under controlled environmental conditions [17,21,22]. SBs are also very often applied to determine the feasibility and actual potential of a biological strategy in the final restoration of a contaminated soil or site [8,11,14]. In fact, under slurry conditions, the pollutant depletion rates depend mainly on the degradation activity of the microorganisms available in the system [20] and the results obtained generally reflect the actual biological depuration potential of the soil [23-28].

The objective of this paper is to present a critical review on slurry bioreactors (SBs) and their application to bioremediation of polluted soils and sediments. The scope of the review encompasses the following subjects: (*i*) process fundamentals of SB and analysis of advantages and disadvantages; (*ii*) the most recent applications of SB to laboratory scale and commercial scale soil bioremediation, with a focus on pesticides, explosives, polynuclear aromatic hydrocarbons, and chlorinated organic pollutants; (*iii*) trends on the use of surfactants to improve bioavailability of contaminants and supplementation with biodegradable carbon sources to enhance cometabolism of pollutants; (*iv*) recent findings on the utilization of electron acceptors other than oxygen; (*v*) advances made in characterization of microbial communities of SB; (*vi*) developments on ecotoxicity assays aimed at evaluating bioremediation efficiency of the process.

## 2. Process fundamentals and analysis of advantages and disadvantages of slurry bioreactors

Slurry bioreactor technology is an engineered complex that generally comprises four parts: installations for polluted soil handling and conditioning, the bioreactor battery itself, installations for treated soil handling and disposal, and ancillary equipment for treatment of process by-streams [18,20]. The SB can be classified as batch, semi-continuous, and continuous from the operation point of view. The most common operational mode is batch. Another useful classification relies on the main electron acceptor used in the biodegradation process: aerobic (molecular oxygen), anoxic (nitrate and some metal cations), anaerobic (sulfate-reducing, methanogenic, fermentation) [13], and mixed or combined electron acceptors [29-31]. By far, aerobic SBs have predominated in full scale applications, although anaerobic SBs are an emerging area of research and development [21].

Interesting and distinctive features of SBs are that soil is treated in aqueous suspension, typically 10 to 30% w/v and that mechanical or pneumatic mixing is provided. These characteristics, in turn, lead to several process advantages, *inter alia*: (*i*) increased mass transfer rates and increased contact microorganisms/pollutant/nutrients; (*ii*) increased rates of pollutant biodegradation compared to *in situ *bioremediation or to *ad situ *solid phase biotreatment: (*iii*) associated to (*i*) and (*ii*), significantly shorter treatment times can be achieved; (*iv*) possibility of using different electron acceptors (O_2_, SO_4_^-2^, CO_2_, NO_3_^-^); (*v*) control and optimization of several environmental parameters such as temperature, pH, etc.; (*vi*) effective use of biostimulation and bioaugmentaion; (*vii*) increase pollutant desorption and availability through the addition of surfactants and solvents [2,10,12,13,32].

However, SBs also have a few disadvantages, all of them related to requirements for soil excavation, handling, and conditioning, and bioreactor construction/operation that typically increase treatment costs compared to most simple bioremediation techniques [20]. In spite of this, SBs has resulted more cost effective than incineration, solvent extraction and thermal desorption in many cases [33].

### 2.1 Process description

**Figure 1:**
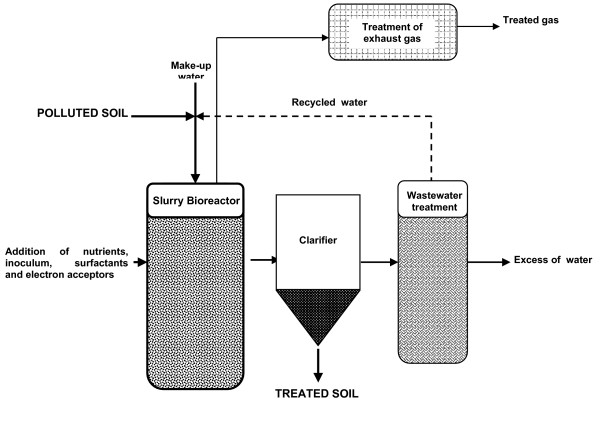
Flow diagram of a typical slurry bioreactor installation. Clarifier is optional.

In SB, soil is excavated and conditioned and loaded into bioreactors [17,34]. A main feature of SB is that soil inside reactor is kept in aqueous suspension by some type of mixing in a way that biological treatment is carried out under saturated conditions [18] and nearly homogeneous suspension [17] (See Figure 1).

There are several bioreactor configurations [20,21]. At full scale, low cost bioreactors may consist of large lined lagoons (24 m × 15 m). Manufactured bioreactors can range 3 to 25 m diameter and 4.5 to 8 m height, with capacities between 60 to 1000 m^3 ^[12,18,19]. Bioreactors are usually fitted with mixing devices, and aerobic SB are equipped with spargers or diffusers. Ancillary equipment may include gas emissions conduits and treatment, vessels for nutrient and pH conditioning of slurry, etc.

Main operating modes of SB include batch and semi-continuous. Continuous SB are possible in principle, but are not very common. Batch and semi-continuous reactors adapt easily to the handling of soils and slurries [18,35], sometimes are operated in sequencing batch reactors [5,20,21].

### 2.2 Soil pretreatment

As it was mentioned above, one of the limitations of SBs technology is the requirement of soil excavation and pre-treatment. Usually, pretreatment consists of crushing followed by screening. The coarser fractions of soils (pebbles and sands, 0.85 to 4 mm) are discarded and sent to direct disposal, whereas fine fractions (clay and organic matter, < 0.85 mm) are retained and loaded into bioreactors. It is generally recognized that pollutants concentrate in fine particles of soil [16,19].

### 2.3 Solids concentration

Polluted, fine fractions of soils are mixed with water or wastewater to form slurry with a concentration in the range between 15 to 60% w/v, depending on characteristics of soil and degradation rates determined in previous laboratory or pilot scale studies [20,21]. Solids concentration is a key variable that might determine the mixing power required, aeration efficiency in aerobic SB, and the size of by-stream post-treatment installations [18]. It is a common practice to use decanted spent liquors from previous batch runs as water for making the slurries, as a method to minimize liquor treatment and disposal.

### 2.4 Mixing

SBs are provided with mixing devices in order to keep solids in suspension during biotreatment, as well as intensifying turbulence and thus increasing mass transfer rates [21]. There are several types of mixing devices, mechanical and pneumatic ones are the predominant among aerobic SBs. Also, mixing can be intermittent or continuous. The first leads to significant savings on power expenses, although mixing intensity is lower.

Mixing intensity is a critical factor in SB design and performance [21]. Its main role is to keep solid particles in suspension and provide slurry homogeneization, to help achieving a satisfactory aeration in aerobic SBs, and to increase several mass transfer rates such as desorption of pollutants from soil, among others. This, in turn, usually translates in higher rates of contaminant biodegradation, in particular when difficult-to-degrade xenobiotic compounds such as PCBs have to be removed [14]. Mixing selection and sizing mainly depends on slurry characteristics and kinetic requirements. Pilot studies and semi-empirical methods are usually required. Independently of the type of mixer selected, mixing power is of major concern because of its influence on the operating costs. The denser the slurry, the higher the power and more difficult to achieve oxygen transfer in aerobic SBs [19,34]. So, several trade-offs have been recommended to keep power expenses at bay, such as intermittent mixing and slurry dilution [18].

### 2.5 Control of environmental conditions in the operation of slurry bioreactors

One distinctive and advantageous feature of SBs is the manipulation and control of environmental conditions that leads to biodegradation optimization and better process performance [17,21,35]. In this regard, several operational variables can be monitored and controlled: pH, dissolved oxygen in aerobic SBs, concentration of inorganic nutrients, pH is usually kept in the range 6.75–7.25 using either alkalis such as NaOH or acids such as H_2_SO_4_. Temperature range is 25–30°C, although SBs have been reported to successfully perform at lower ambient temperatures. Ideal dissolved oxygen concentration is 90% of the saturated dissolved concentration. Nitrogen and phosphorus salts are used as inorganic nutrient sources in order to ensure that there is no nutrient limitation (NH_4_Cl, KH_2_PO_4_, Na_2_HPO_4_) [5,17,36].

## 3. Applications of aerobic slurry bioreactors to bioremediation of soils polluted with pesticides, organo-chlorinated substances, explosives, and polynuclear aromatic hydrocarbons

Application of aerobic SBs (A-SB) to bioremediation of soils is still predominant. Aerobic degradation of organic pollutants is carried out by its oxidation via oxygenases or dioxygenases, so, molecular oxygen is usually a limiting factor [37]. A large number of laboratory, pilot and full scale studies and cases of A-SB can be found in the literature. In Tables 1, 2 and 3 we have excerpted some applications of A-SB to bioremediation of soils polluted with PAHs, pesticides and PCBs, and explosives, respectively.

**Table 1:** Remediation of sites/actual site soils contaminated with PAH in aerobic slurry bioreactors.

**Matrix**	**Contaminant**	**Remarks**	**Removal**	**Ref.**
Soil	PAH^a^: Pyrene	a) without surfactant b) Brij 30	a) 63% b) 70%	[106]
Soil	PAHs^a^	- *Corynebacterium aquaticum* - *Flavobacterium mizutaii* - *Mycobacterium gastri* - *Pseudomonas aeruginosa* - *Pseudomonas putida* - Biosurfactant	93%	[92]
Soil Sandy	PAHs^a^: a) Pyrene (358 mg/kg), b) chrysene (255 mg/kg), c) benzo [*a*]pyrene (250 mg/kg)	- PAH-degrading consortium - 30% (v/v) silicone oil	a) 19 mg L^-1 ^day^-1^ b) 3.5 mg L^-1 ^day^-1^ c) 0.94 mg L^-1 ^day^-1^	[40]
Soil	PAHs^a^	a) Clay soil b) Sand soil	a) 43% b) 25%	[41]
Estuarine sediments	PAH^a^ Phenanthrene	a) Mixed system b) Unmixed system - 7 days incubation - 5, 10, 15% sediment load	a) 25 – 40% b) 5 – 20%	[38]
Soil	PAH^a^ Naphthalene	*Pseudomonas putida*	100%	[107]
Waste sludges	PAHs^a^ Pyrene, benzo [*a*]anthracene, chrysene	Petroleum waste sludges	90%	[33]
Soil Clay 20% Sand 79% Organic matter 1%	PAH^a^ a) Naphthalene (500 mg/kg) b) Naphthalene (5000 mg/kg) c) Naphthalene (25000 mg/kg)	*Flavobacterium sp.*	a) 96% b) 99% c) 99%	[9]
Actual site soil	Complex mixture of PAHs^a^(~13.0 g/kg)	Slurry-phase in the absence and presence of soya lecithin or humic substances at 1.5% w/w	25% (without agents) 58% (with agents)	[11]
Actual site soil	Complex mixture of PAHs^a ^(~3.7 g/kg)	Comparison SB, blade agitated bioreactor and rotary vessel bioreactor	SB was capable of the readiest and fastest removal of soil PAHs	[8]
Soil	PAH^a ^Fluorene	*Absidia cylindrospora *Maltosyl-cyclodextrin	90%	[108]
a) Pristine sediment Clay 9% Silt 16% Sand 75% Organic matter 1.7% b)Contaminated sediment Clay 6% Silt 5% Sand 89% Organic matter 5.1%	a) supplemented with PAHs^a^: phenanthrene, anthracene, pyrene and fluoranthene (60–70 ppm) b) PAHs^a^: phenanthrene (36.5 ppm) anthracene (110.2 ppm) pyrene (350 ppm) fluoranthene (150 ppm)	*Rhodococcus sp.*	a) 100% b) >98%	[39]

Notes: ^a^PAHs: Polynuclear aromatic hydrocarbons

**Table 2:** Remediation of sites/actual site soils contaminated with pesticides and PCBs in aerobic slurry bioreactors.

Matrix	Contaminant	Remarks	Removal	Ref.
Waste activated sludge	EPTC^a ^(5 – 6 ppm)	a) freely suspended activated sludge b) immobilized activated sludge	a)35% b)72%	[109]
Agricultural soil Clay 56.11% Silt 23.2% Sand 20.69% pH 8.3 Organic carbon 0.6%	γ-HCH^b^	Inoculated with *Pseudomonas putida *coexpressing cytocrome P-450 cam and luciferase	65%	[48]
Soil Clay 13% Silt 21% Sand 66% pH 7.7 Total carbonates 3%	a) PCBs^c ^(1210 mg/kg) b) TPH^d ^(11407 mg/kg)	Rhamnolipid production was observed by *Pseudomonas aeruginosa*	a) 98% b) 99%	[49]
Actual site contaminated soil	PCBs^c ^(1547 mg/kg)	Inoculated with ECO3 co-culture	21% (without inoculum) 39% (with inoculum)	[47]
Actual site contaminated Soil	PCBs^c ^(1547 mg/kg)	Inoculated with ECO3 co-culture; optimization of SR configuration	18% (in shake flasks with baffles) 30% (in stirred thank reactor)	[14]
Actual site contaminated Soil	PCBs^c ^(350 mg/kg)	Inoculated with ECO3 co-culture in the absence and the presence of Cyclodextrins, Quillaya Saponin, Triton X-100	65% (without agents) 80% (with agents)	[46]
Soil Clay 48% Silt 41% Sand 11% pH 7.0 Organic matter 4%	2,4-D^e ^(300 mg/kg)	With and without sucrose	>90%	[30]
Soil Clay 26% Silt 48% Sand 26% pH 7.2 Organic matter 0.8%	DEP^f ^(8 mg/g)	a) with native soil microflora b) with effluent treatment plant microflora	a) 47.9% b) 75.4%	[22]

Notes: ^a^S-ethyl dipropylthiocarbamate; ^bγ^-hexaclhorocyclohexane; ^c^Polychlrorinated byphenyls; ^d^Total petroleum hydrocarbons; ^e^2,4-dichlorophenoxyacetic acid; ^f^di-ethyl phthalate

**Table 3:** Remediation of sites/actual site contaminated with explosives in aerobic slurry bioreactors.

Matrix	Contaminant	Remarks	Removal	Ref.
Soil from Joliet Army Ammunition Plant	2,4,6-TNT ^a^ 2,4,6-TNB^b^	Addition of molasses	90%	[12]
Soils from Army Ammunition Plant a) soil VAAP moisture 3% bulk density 1.4 g/ml pH 4.6 b) soil BAAP moisture 1.6% bulk density 1.7 g/ml pH 9,35	a) 2,4-DNT ^b ^(14715 mg/kg) 2,6-DNT^c ^(8940 mg/kg) b) 2,4-DNT ^b ^(1125 mg/kg) 2,6-DNT^c ^(4800 mg/kg)	Augmentation with a DNT-mineralizing culture	98%	[5]
Soil	2,4,6 trinitrophenylmethylnitramine^d^	-	99%	[110]
Soil	2,4,6-TNT^a^	-	40%	[111]

Notas: ^a^2,4,6-trinitrotoluene; ^b^2,4,6-trinitrobenzene

Jee *et al. *[38] studied the bioremediation of sediment contaminated by 88 mg/kg phenanthrene. The influence of different solid/sediment loads and mixing conditions on the time course of the process was investigated. Under well mixed conditions, phenanthrene was removed by 25 to 40% during the first few days of treatment by showing an activity which decreased markedly from the 3rd to the 5th day. In unmixed systems, the extent of mineralization was notably lower, ranging from 5 to 20%. The pollutant mineralization did not cease by end of the 7th day of treatment. Under well mixed conditions, the reactors with a 10% sediment load showed the highest level mineralization. Pollutant mineralization yields achieved in the reactors with 5 and 15% sediment were similar, and lower than those attained with 10% sediments. In the unmixed reactors, the 10 and 15% solids loaded reactors showed similar activity.

Dean-Ross [39] conducted a A-SB bioremediation batch experiment on a pristine sediment spiked with phenanthrene, anthracene, pyrene and fluoranthene. PAHs, each added at 60–70 mg/L, disappeared after 6 to 8 days of treatment. Bioaugmentation with *Rhodococcus sp*. resulted in enhanced bioremediation rates and yields.

Villermur *et al. *[40] used a three 3-phase (two liquid phases + soil, TLP) aerobic slurry system containing silicone oil at 30% v/v to biodegrade selected high molecular weight PAHs spiked into a soil at 196 mg pyrene/kg, 192 mg chrysene/kg, and 163 mg benzo [a]pyrene/kg. Pyrene was degrade d through a rate of 19 mg/(L day) whereas the degradation rates of chrysene and benzo [a]pyrene were, 3.5 and 0.94 mg/(L day), respectively.

Wang & Vipulanandam [9] observed the effect of naphtalene concentration on the A-SB remediation potential. Naphthalene was generally found to rapidly desorb from the spiked soil by undergoing rapid and extensive biodegradation. 500 mg/kg of naphthalene were reduced to 20 mg/kg after 65 h days of treatment whereas 5000 mg/kg of the same pollutants were reduced to 40 mg/kg after 100 h.

Castaldi [33] treated petroleum waste sludges containing 680 mg/Kg of 2–3 ring PAHs and 38 mg/kg of 4–6 ring PAHs in a continuous flow multistage A-SB that operated at relatively short residence times with minimal loss of volatiles constituents. The higher molecular weight PAHs, including the four-ring derivatives of pyrene, benzo [a]pyrene, and chrysene, were removed with efficiencies greater than 90%.

Rutherford *et al. *[41] treated in a A-SB two different soils (a sandy soil and a silty clay one) contaminated with creosote compounds in the range 10 000 to 20 000 mg/kg. Unexpectedly, the extent of PAHs removal over 10 weeks was higher in the clay soil (43%) than in the sandy one (25%).

De Jonge & Verstrate [42] reported 70% removal from a soil initially polluted with up to 3 000 mg/kg of oil in A-SBs. Also, soils heavily contaminated with crude oil (200 g TPH/kg) could be treated in pilot A-SBs reaching 1 to 2 g TPH/kg in 5–7 weeks of treatment [43].

Castaldi & Ford [15] reported 73% removal of PCBs in 90 days of A-SB treatment of petrochemical waste sludges polluted with 115 mg/kg of PCBs. Bioremediation of sediments contaminated with 88 mg phenanthrene/kg in batch A-SB showed removal percentages of 25–50% in 7 days of operation [36]. On the other hand, Mueller *et al. *[17] observed the extensive removal (up 98%) of pentachlorophenols and creosote from soils and sediments in 30 days of operation. A soil spiked with a mixture of PAHs (270 mg/kg), composed by fluorene, phenanthrene, anthracene and pyrene, was treated in a batch A-SB for 20 days: fluorene and phenathrene were found to be removed by 70% and 40% after 3 days of incubation [16]. In another work with soil polluted with PAHs, Lewis [44] reported a removal of 70–97% of spiked PAHs (728 – 4 920 mg/kg) in 60 days of treatment.

Pinelli *et al. *[8] studied the intrinsic depuration capability of a soil historically contaminated by PAHs (overall initial PAHs: 3.7 g/kg) by using different aerobic batch bioreactors: a SB, a blade agitated bioreactor and a rotary vessel bioreactor. The performance of each bioreactor was evaluated by analyzing the disappearance of 14 target PAHs and of the total extractable organic matter of the soil. About 72, 96 and 95% of the total amount of PAHs initially present in the soil were removed in the SB, blade agitated semisolid-phase bioreactor, and rotatory vessel semisolid-phase bioreactor after 17, 47, and 35 days of treatment, respectively. Among the three treatments, SB was capable of inducing the readiest and fastest removal of the soil PAHs.

Fava *et al. *[11] treated an actual site historically contaminated soil containing about 13 g/kg of a large variety of PAHs in laboratory scale A-SB amended with soya lecithin or humic substances at 1.5% w/w. After 150 days of incubation of a room temperature, about 60% of original amount of PAHs was biodegraded with a marked reduction of the original soil ecotoxicity.

A-SBs have also been successfully applied to bioremediation of soils contaminated with pesticides. Cookson [20] reported that levels of pesticides in soils polluted with mixtures of 2,-4-dichlorophenoxy acetic acid (2,4-D), 4-chloro-2-methyl-phenoxy acetic acid, alachlor, trifuralin and carbofuran could be reduced from 800 to 20 mg/kg after two weeks of treatment. Robles-González *et al. *[30] carried out laboratory scale experiments with batch A-SB for bioremediation of an agricultural soil with high contents of clay and organic matter and polluted with 300 mg 2,4-D/kg. They observed removals of 2,4-D higher than 95% after 14 days of treatment; no chlorophenol intermediates of 2,4-D degradation were detected. Bachman *et al. *[27] reported 90–100% mineralization of α-hexachlorocyclohexane (HCH) from a soil initially polluted with 400 mg α-HCH/kg after 42 days of batch incubation of laboratory scale A-SB.

SBs also provided excellent results in the aerobic bioremediation of actual site PCBs-contaminated soils. Fava and co-workers [45,46] reported of the efficient bioremediation of an actual site soil with 350 mg/kg of total PCBs through 141 days of A-SB treatment. They also demonstrate the possibility of enhancing the process through the SB supplementation with cyclodextrins (CDs) or the phytogenic surfactant Quillaya Saponin at 10 g/L. The opportunity to improve the aerobic slurry-phase bioremediation of another actual site soil lacking indigenous specialized bacteria and contaminated by 1547 mg/kg of PCBs through (a) soil bioaugmentation with the PCB mineralizing three-member bacterial coculture ECO3 [47] or (b) improvement of SB configuration [14] was successively investigated and demonstrated by the same authors.

Rattan *et al. *[48] found that batch soil slurry microcosms inoculated with a strain of *Pseudomonas putida *coexpressing cytochrome p-450 cam and luciferase, removed more than 65% of γ-HCH (105 mg/L slurry) after 4 weeks of incubation. On the other hand, less than 15% was the γ-HCH depletion achieved in the parallel non-inoculated controls.

Hudak & Cassidy [49] treated an aged contaminated soil in SB; lubricating oil (LO) and polychlorinated biphenyls (PCBs) were the pollutants applied at the concentration of ~11 400 mg/kg and ~1200 mg/kg, respectively. They also examined biosurfactant production by *Pseudomonas aeruginosa*. Rhamnolipid production was observed within 1 to 2 days after nitrogen depletion. By day 6, total rhamnolipid concentrations increased from below detection to average values over 1 000 mg/L, which was associated to removals over 98% of soil-bound PCBs and over 99% of total petroleum hydrocarbons.

Mohan *et al. *[22] studied the degradation of a di-ethyl phthalate (DEP) contaminated soil in a sequencing A-SB; the effect of bioaugmentation using effluent treatment plant (ETP) microflora on the degradation of DEP was also investigated. Consistent desorption of DEP was observed in the aqueous phase with increase in the contact time (cycle period) due to prevailing agitation in the reactor. Reactor operated with native soil microflora (7.6 × 10^3 ^CFU/g) and exhibited a maximum DEP degradation of 47.9%. Initially, up to 8 h of the cycle operation, the degradation of DEP in soil was found to be 9.85% and reached a maximum of 47.9% in 40 h. Another reactor was operated in the presence of ETP-degrading microflora (2.4 × 10^7 ^CFU/ml) without native soil microflora. Within 8 h of cycle operation a DEP degradation of 22.45% was noticed and reached a maximum of 75.4% at the end of cycle period. It was concluded that bioaugmentation with ETP-acclimated biomass significantly increased DEP removal.

Zhang *et al. *[5] have conducted pilot scale studies on bioremediation with A-SB of soils contaminated with explosives. They treated two soils from a former ammunition manufacturing plant, polluted with 14715 mg/kg to 1125 mg/kg of 2,4-dinitrotoluene (2,4-DNT) and 8940 mg/kg to 4800 mg/kg of 2,6-dinitrotoluene (2,6-DNT). Almost complete removal of explosives could be achieved in a short 2 days period. Moderate removal of 40% was reported for a soil polluted with 570 mg/kg of 2,4-DNT and 380 mg/kg of 2,6-DNT [50]. Laboratory scale experiments with A-SB showed an effective removal of explosives (90%) from soils with initial 400–1200 mg TNT/kg after 130 days of treatment [12].

## 4. Utilization of electron acceptors other than oxygen

Recent research has shown that non aerobic bioremediation holds promise for remediation of specific recalcitrant and toxic contaminants (such as organo-chlorinated pesticides and compounds, nitro-organics, etc.) where soils are rich in sulfate, nitrate, or bicarbonate [13,30,51-54].

Very often, non aerobic bioremediation is also preferable when anaerobic or reducing conditions predominate in the contaminated sites. In such scenarios, anaerobic bioremediation may be desirable whenever the pollutants are amenable to reductive dechlorination or nitro-reduction as first step to final biodegradation. Another advantage of anaerobic remediation is associated to savings of aeration (both investment and operating costs of aeration) [29,30].

Reported studies on anaerobic bioremediation of soils and/or sediments in SB are still scarce. Table 4 summarizes a selection of recent research published in the open literature. Boopathy [29] treated a soil contaminated with 550 mg diesel/kg in several anoxic and anaerobic batch, laboratory scale SB. He found the highest removal of 80% in a SB with "mixed" or combined electron acceptors (SO_4_^=^, NO_3_^-^, CO_2_). One electron acceptor-SB showed lower removals, for instance, 55% of diesel removal was observed in the sulfate-reducing SB. Soils polluted with 1500 mg TNT/kg have been treated in batch, pilot scale anaerobic SB; up to 95% removal of TNT was reported [55].

**Table 4:** Anaerobic slurry bioreactors.

Matrix	Contaminant	Remarks	Removal	Ref.
Soil	2,3,7,8-tetrachlorodibenzo-*p*-dioxin (TCDD)	With anaerobic activated sludge as the microbial inocula and sludge cakes as the primary substrates	86%	[112]
Soil	Chlorpyrifos	Sequencing batch mode (anoxic-aerobic-anoxic)	91%	[113]
Soil	Hexahydro-1,2,3-trinitro-1,3,5-triazine (RDX)	With supplementation of municipal anaerobic sludge as an exogenous source of microorganisms	60%	[114]
sediment	PAH Acenaphthene	Addition sulfate as an electron acceptor enhanced PAH degradation	79%	[115]
Soil	αβδ and γ-hexachlorocyclohexane	Bioaugmented with anaerobic sludge	100%	[68]
Soil	2-sec-butil-2,6-dinitrofenol (DINOSEB)	Pilot scale	51%	[55]
Soil	2,4-dichlorophenoxy-acetic acid	Sulfate reduction conditions with addition of sucrose	48%	[30,31]
Soil	Pentachlorophenol (PCP)	Bioaugmented with cells of *Desulfitobacterium frappieri *strain PCP-1	100%	[87]
Soil	Trinitrotoluene (TNT)	Augmentation with anaerobic biomass from a food industry wastewater treatment plant	100%	[116]
Soil	Diesel	a)mixed electron acceptor (SO_4_^=^, NO_3_^-^, CO_2_) b)sulfate-reducing condition	a)80% b)55%	[29]
Soil	Tetrachloroethylene	Bioaugmented with cells of *Desulfitobacterium sp. s*train Y-51 and addition of zero.valent iron (Fe^0^)	100%	[117]

Bachman *et al. *[28] tested aerobic and several laboratory scale anaerobic SB for the bioremediation of a coarse soil polluted with 400 mg α-HCH/kg. They reported 90 to 100% mineralization of the pesticide after 100 days of treatment in the A-SB, 85% in the methanogenic SB, whereas in the sulfate-reducing and denitrifying SB no conversion of HCH was observed. A 95% removal of Dinoseb (2-sec-buthyl-2,6-dinitrophenol) in methanogenic conditions was obtained from a soil contaminated with 800 mg/kg of this herbicide [55]. Robles-González *et al. *[30] conducted laboratory scale experiments with batch sulfate-SB for bioremediation of an agricultural soil with high contents of clay and organic matter and polluted with 300 mg 2,4-D/kg. They observed removals of 2,4-D near to 50% after 30 days of treatment and they detected chlorophenol intermediates of the anaerobic transformation of 2,4-D.

## 5. Use of surfactants and solvents to improve availability of contaminants

### 5.1 Advances on hysteresis characterization

Hysteresis coefficient is used for determining the adsorption-desorption behavior of pollutants on sediments and soils. In soil and sediment pollution, the adsorption and desorption play a key role on pollutants availability and transport. For several pollutants and solid matrices, the desorption pathway is different from that of adsorption. This phenomenon is known as hysteresis. Poggi-Varaldo *et al. *[56] have defined a hysteresis coefficient CH as the ratio of the slope (derivative) of the adsorption curve and the slope of the desorption curve in a given point (C_j_, q_j_) of interest. When hysteresis is not important, CH ≅ 1, *i.e., *the adsorption is reversible, when hysteresis is important, then CH >1, *i.e., *the adsorption is irreversible. Among several uses of the CH, it can be cited the following: quantification of the effect of aging on the availability of a pollutant adsorbed onto sediments or soils; quantitative comparison of the degree or retention of a pollutant in several types of solid matrices; quantification of the effect of adding surfactants and solvents to a given system pollutant-solid matrix; quantitative comparison of the degree or retention of several pollutants in a given sediment or soil; and estimation of the retardation factor of transport of contaminant in porous, adsorbent media [31,56].

The availability enhancement factor (AEF) is used for determining the effectiveness of desorption treatments of pollutants from soils and sediments. The AEF was defined as the ratio of the slope of the desorption curve of a given pollutant with a surfactant or solvent treatment to the slope of the corresponding curve of the reference treatment (usually desorption with distilled water). This factor has proved to be useful for the quantification of the effect of a given soil treatment (with surfactant, biosurfactant, solvents) on the possible improvement of the pollutant desorption and availability for further biodegradation or physicochemical removal [57].

### 5.2 Surfactants

Surfactants may facilitate hydrophobic pollutant desorption from a solid matrix, and its dispersion in the aqueous phase. They may also help in stabilizing the slurry soil-aqueous medium in the SB. Surfactants are classified as anionic (SDS, LASA and SDOSS), cationic (benzyl trimethyl-ammonium bromide) and non ionic (Triton X-100; Brij 35; Tergitol NP-10, Tween-80, etc.) from the point of view of predominant electric charge after contact with water [58,59]. They can also been classified as synthetic and biological surfactants, according to its origin or production. The first ones are mainly manufactured by petrochemical plants, whereas the second ones are produced by living organisms. Examples in the second category are rhamnolipids, glycolipids or lipoproteins, among others [60-65].

Main factors that determine surfactant success and capability are its chemical nature and concentration as a multiple of critical micellar capacity (CMC). For synthetic surfactants, it has been observed that although they may be very effective for increasing the detachment of pollutants from soils, they usually become toxic to microbes at the high concentrations required for their action; very often pollutant degradation is adversely affected [35,36,64,66,67]. Results on surfactant capability and performance in the literature has been obscured by experimental designs that have not taken into account the surfactant CMC [58]. In this way, several works report no effect or negative effect of surfactants on pollutant mobilization and detachment from the solid matrix. Closer examination of a large amount of these experiments have revealed that surfactants were used at concentrations lower than their corresponding CMCs. Now it is generally recognized that surfactants should be added at a concentration higher than its CMC in order to effectively disperse and pseudo-solubilize the hydrophobic pollutant in water.

Regarding synthetic surfactants, the order of preference generally accepted is: non ionic > anionic > cationic. Cationic surfactants are usually toxic to microorganisms and also they can adsorb to negatively-charged sites of the clay and organic matter of the soil, reducing the actual concentration value. So, it is recommendable not only to carry out *in vitro *studies for determining surfactant capability [57] but inhibition studies of the candidate surfactants on the active microflora of the SB as well [68].

Biodegradable surfactants and biosurfactants may be the choice for increasing pollutant availability while minimizing inhibitorial effects on SB microflora. Yet, some biodegradable surfactants can compete with the pollutant as carbon source for the microorganisms provoking a preferential degradation of the surfactant and a lower removal of the contaminant [35,36,67,69]. Microbial surfactants have proved to be able to combine excellent hydrophobic pollutant-mobilizing properties with other special features, rarely displayed by commercial chemical surfactants, such as complete biodegradability and non-toxicity [62,65]. Unfortunately, little is very often known about their chemical-physical properties, and they are still not available on the large scale at low price, and this currently precludes their application in bioremediation. It has been recently shown that some commercial mixtures of biogenic compounds, such as cyclodextrins, phytogenic surfactants of water-soluble fraction of humic substances, can markedly intensify the bioremediation of PCB-, PAH- and/or hydrocarbon-contaminated soils in SBs (Tables 1 and 2) by displaying the same advantages showed by microbial surfactants. In particular, Fava and co-workers [45,46] demonstrated that two cyclodextrins (CDs), i.e., hydroxypropyl-β-CD and γ-CD, and the phytogenic surfactant Quillaya Saponin were able to enhance from 15 to 30% the aerobic bioremediation of an actual site, aged PCB-contaminated soil (with 350 mg/kg of total PCBs) when applied at 10 g/l under slurry-phase conditions. The agents did not exhibit the toxicity and the recalcitrance displayed by the chemical surfactants Triton X-100 tested at the same concentration in identical parallel laboratory-scale SBs. Then, the same group [70-72], investigated the opportunity to use less costly industrial CD products, and in particular a mixture of RAndomly MEthylated-β-cyclodextrins (RAMEB), to intensify the aerobic bioremediation of 5 other different PCB-contaminated soils under several treatment conditions including laboratory-scale SBs. A faster and more complete removal of PCBs was generally observed in the amended bioreactors for the ability of RAMEB to improve both PCB occurrence in the soil water-phase and the growth and the occurrence of soil specialized bacteria in the reactors. Larger PCBs biodegradation and dechlorination, along with larger toxicity depletion, were generally observed in SBs than in the solid-phase or saturated soil loop reactors, especially in the presence of RAMEB. This may be ascribed to the higher degree of homogeneity and higher mass-transfer rates typical of slurry-phase conditions [20,21] which, in turn, probably enhance the intimate contact among the specialized microorganisms, the mobilizing materials and the soil-sorbed PAHs. Then, the beneficial effects of RAMEB have been also demonstrated in the aerobic bioremediation of PAH- and transformer oil-contaminated soils under laboratory slurry and solid-phase conditions as well as in the field, through pilot scale experiments conducted both under *ex-situ *(biopile) and *in situ *conditions [73]. Recently, the potential enhancing effects of other two commercial biogenic pollutant-solubilizing agents, namely a technical mixtures of Soya Lecithins and an extract of water soluble humic substances of North Dakota Lignite, on the aerobic slurry-phase bioremediation of a model soil spiked with PCBs [74,75] and of an aged PAH-contaminated soil [11] have been demonstrated. In the latter case, a soil historically contaminated by about 13 g/kg of a large variety of PAHs was amended with soya lecithin or humic substances at 1.5% w/w and treated in aerobic solid-phase and SBs for 150 days. The overall removal of PAHs in the presence of the agents was faster and more extensive and accompanied by a larger soil detoxification, especially under slurry-phase conditions. The agents could be metabolised by soil aerobic microorganisms and enhanced the occurrence of both soil PAHs and indigenous aerobic PAHs-degrading bacteria in the reactor water-phase. Thus, the agents were biodegradable and efficiently enhance PAH biodegradation by improving the availability of both PAHs and specialized microorganisms in the soil reactors.

All biogenic pollutant-mobilizing agents mentioned above were found to be promising bioremediation enhancing additives, as capable of combining marked enhancing effects on the process with a complete biodegradability, non-toxicity and therefore a complete environmental compatibility. In addition, they are biogenic compounds and this makes their incorporation into soil more socially acceptable than that of the chemical surfactants currently proposed to intensify the bioremediation of aged PCB- and hydrocarbon-contaminated soils.

### 5.3 Solvents

Solvents can also be used for increasing the availability and bioavailability of low solubility-, hydrophobic pollutants in soil remediation. In particular, immiscible (in water) and non degradable solvents with affinity for hydrophobic contaminants can help in attracting the molecules of contaminants adsorbed onto soil, transfer the contaminant into the solvent phase, and afterwards to facilitate the exchange of contaminant between the solvent to the aqueous phase [6,76] where microorganisms can finally degrade the pollutant. In SB practice, there is little or no experience on using solvents for this purpose. If it were done, the SB would be a tri-phasic bioreactor (3P-SB) with one particular solid phase (soil and microorganisms) and two liquid phases (droplets of solvent dispersed in the water phase).

Available information mainly comes from the application of two-phase 2P (liquid, solvent-water) bioreactors (Table 5). Knowledge accumulated from the 2P (liquid) bioreactor practice indicates that best solvents used for enhancing biodegradation of PAH are paraffin oil, silicone oil, n-hexadecane, corn oil, cis-jasmone, r-limonene, 2-undecanone, l-decene and n-dodecane [6,64,77]. The 2P bioreactor concept has been shown to be very effective for biodegradation of high concentrations of toxic organic compounds by bacteria, Table 5.

**Table 5:** Biphasic and triphasic bioreactors for treatment of hydrophobic organic pollutants (adapted from [78]).

**Pollutant**	**Microorganisms**	**Solvent**
Styrene	Mixed culture	silicone oil
Phenantrhene	*Pseudomonas aeruginosa*	2,2,4,4,6,8,8-heptametilnonane
Naphthalene	*Corynebacterium sp.*	Decane, dodecane, hexadecane
Various PAHs	Mixed culture	silicone oil
2,4,6-trichlorophenol	*Pseudomonas *sp.	silicone oil
Dioxins	Mixed culture	Decane
Pentaclorophenol	*Arthrobacter *sp.	Diethyl sebacate
Phenol	*Pseudomonas putida*	2-undecanone
Phenanthrene	*Pseudomonas *sp.	silicone oil
Benzene	*Alcaligenes xylosoxidans*	Hexadecane
Benzene	*Klebsiella *sp.	1-octadecane
Benzene	*Alcaligenes xylosoxidans*	Hexadecane

For successful application of either 3P or 2P-SB technology, appropriate selection of the solvent is of paramount importance: solvent should be immiscible in water, non toxic to microorganisms, non biodegradable, stable and non flammable, non volatile, and with high affinity for hydrophobic pollutants [78]. According to Villemur *et al. *[38] and Collins & Daugulis [79] 2P bioreactors might provide an efficient way to bioremediate contaminated soils. For example, in a large scale process, a 2P-SB (soil-solvent) could be used to extract pollutants from a contaminated soil by transferring to the water-immiscible, non degradable liquid phase. The solvent would then be recovered and the treated soil returned to the site. This could be repeated a number of times with the same solvent. Once the water-immiscible liquid has reached a certain concentration of contaminant, the extraction vessel could then be used as a 2P-bioreactor (liquid, solvent and water) to degrade the extracted contaminants.

## 6. Bioaugmentation and advances on characterization and monitoring of microbial communities of slurry bioreactors

Very often SB operation relies on the use of native microflora already existing in the polluted soil. However, whenever native microflora is scarce or weak or with no apparent capability of degrading the target compounds, it is advisable to inoculate SB with enriched/acclimated consortia or strains, more commonly consortia. This is called bioaugmentation. Thus, inoculation of 'specialized' biomass may allow for an increased biodegradation of target pollutants as well as a more effective detoxification of the solid matrix [47,80,81]. Another common result of bioaugmentation is the dramatic reduction of remediation times [5].

Barbeu *et al. *[82] have recommended a protocol for producing aerobic acclimated consortia based on activated sludge process. An outline of their procedure follows: start a complete-mix activated sludge reactor with 10% (w/v) of soil and a mineral medium. The concentration of the target pollutant(s) in the feed of bioreactor is cautiously increased step-wise until the desired concentration in the feed is reached. After 30 days of operation, provided that reactor monitoring indicates the uptake of the contaminant, an acclimated consortium is developed with the capability of using the target pollutant(s) as energy and carbon sources. Acclimated inocula can also been obtained from polluted sites such as sediments, soils and wastewaters [6,9,64]

To a lower extent and mainly in laboratory and pilot scale trials, specific strains have been used for bioaugmentation. A three-membered co-culture of strains of *Pseudomonas *sp and *Alcaligenes *sp. have been applied to aerobic degradation of PCBs [47]; *Sphingomonas aromaticivorans *to degradation of PAHs [6]; *Flavobacterium *sp. for degradation of naphtalene [9]*Burkholderia cepacia *JS872, *B. cepacia *and *Hydrogenophaga palleronii *for degradation of 2,4-DNT and 2,6-DNT [5]. However, several failures have been recorded in this field; they have been mostly ascribed to the inability of inoculated specialized pure or mixed cultures to compete with autochthonous microflora and to face the toxicity and the scarcity of nutrients occurring in the contaminated biotope [83]. Thus, the opportunity to bioaugment contaminated soils with complex microbial systems consisting of a large variety of robust microorganisms and essential nutrients has been recently explored [33,84]. In particular, Di Toro *et al. *[85] studied the effects of Enzyveba, i.e. a partially characterized consortium of microorganisms developed from the stabilization of high quality organic wastes, on the aerobic bioremediation of an actual-site aged PCB-contaminated soil (overall PCBs: 920 mg/kg) under laboratory-scale slurry and solid-phase conditions. Markedly enhanced PCB-biodegradation rates and extents (from 50 to 100%) and soil detoxification as well as higher availability and persistence of aerobic PCB- and chlorobenzoic acid-degrading cultivable bacteria were observed in the Enzyveba-amended SBs. These findings suggested that Enzyveba enhanced the biotreatability of the selected soil by providing exogenous bacteria and fungi able to remove inhibitory or toxic intermediates of PCB biodegradation and/or exogenous nutrients able to sustain microorganisms in charge for PCB mineralization.

Also, dehalorespiring strains that use organo-chlorinated compounds as electron acceptors have used in bioaugmentation such as *Desulfitobacterium frappieri *PCP-1 and *Desulfitobacterium dehalogenans *introduced into non-sterile soil or soil slurry microcosms [86,87].

Monitoring of microbial communities and specific strains in soil SBs is becoming an important tool for improving our understanding of microbial population dynamics [88], the fate of inoculated cultures as well as biodegradation mechanisms, and the influence of process operating conditions and other variables [87].

Monitoring of microbial community is traditionally accomplished by microbial counting by total or selective plating, most probable number counting, or bacterial staining followed by microscopic analysis, or by measuring microbial activity through respirometric or radiorespirometric analyses [89]. Due to experimental limitations, such as the lack of suitable electron donors or acceptors or of nutritional factors in the growth media or the unsuitable growth conditions as regards temperature, pressure or redox conditions, most of these methods are inadequate to give a reliable picture of the microbial community. However the most important factor affecting the reliability of methods based on cultivation of microorganisms is the poor culturability of most of microorganisms in complex environments such as those created in the laboratories [90]. When the active microbial population is poorly cultivable the effectiveness of a remediation process cannot be efficiently predicted or followed during a treatment by using traditional microbiological methods. Modern molecular biology tools offer in these cases several advantages respect to traditional microbiological methods, since they can couple qualitative and quantitative information on the microbial community including the uncultivable fraction. The polymerase chain reaction (PCR) and competitive PCR (cPCR) have been used with success to monitor *Desulfitobacterium frappieri *PCP-1 and *Desulfitobacterium dehalogenans *introduced into non-sterile soil or soil slurry microcosms [86,87]. A previous report showed that strain PCP-1 can dechlorinate PCP in anaerobic soil slurry microcosms and can be monitored by PCR when introduced in this system [91]. The fatty acid methyl ester (FAME) analysis approach was successfully employed by Cassidy & Hudak [92] to selectively monitor the main bacterial and yeast strains responsible for biosurfactants production in a 8 L slurry-sequencing batch reactor developed to treat a soil contaminated with 21.8 g/kg diesel fuel. The results of the study demonstrated that SB operation can be manipulated on the basis of microbial monitoring information to control and improve the overall reactor performance [92]. No other reports on microbial molecular/biochemical monitoring of SBs were found in the literature. However, such approaches might allow predicting, following and improving the effectiveness of SB bioremediation processes, and therefore they should be included in the integrated strategies for SB monitoring and assessment.

## 7. Developments on ecotoxicity assays aimed at evaluating bioremediation efficiency of the process

Several types of ecotoxicity bioassays are known, some of them have already been standardised, some others are under development [93,94]. They are carried out in polluted and treated soil to further assess treatment performance and end point. Information of these tests shed light on "true" soil detoxification achieved by treatment, in opposition of "apparent" decontamination assessed by mere chemical or instrumental analysis. In effect, pollutant removal sometimes may be accompanied by transformation of the mother contaminant into toxic intermediates that could persist in soil. So, contaminant removal not always is equivalent to decontamination or detoxification [95]. Ecotoxicity tests also account for the interactions among pollutants, which might interact through a synergic or antagonistic manner. Again, this type of interaction cannot be observed by mere instrumental analysis [96]. Thus, ecotoxicity assays appear as a significant complementary tool to assess the extent and success of bioremediation [97].

Some ecotoxicity tests are carried out using the aqueous extracts of soils: they are useful when pollutants are water soluble. The OECD [98-102] has proposed to apply a variety of tests organisms belonging to different levels of the trophic chain: microorganisms (*Pseudomonas putida, Photobacterium phosphoreum*), primary consumers (micro crustaceans Daphnias); secondary consumers (fish); primary producers (algae).

Yet, some other tests are performed using whole soil. These methods are also quoted as direct contact tests, as they rely on the growth or germination of well defined, artificially cultivated test-organisms directly into the contaminated soils. Test organisms include plants (*Brassica rapa, Avena sativa, Lepidium sativum*, etc), seeds for assessing germination, and mortalitiy of earth worms (*Eisenia fetida, Eisenia andrei*) [103]. These tests are particularly indicated for those soils contaminated by hydrophobic pollutants such as PAHs and PCBs, which typically are sorbed onto the soil. Other ecotoxicity assays rely on the evaluation of biological abundance or diversity of organisms autochthonous of the contaminated soils (nematodes, microorganisms) [97,100,104] or in the measure of some specific activities of the soil, like respiration, enzyme activities, etc. [105].

A combination of the *Lepidium sativum *roots and shoots elongation inhibition test and of *Folsomia candida *mortality test has been successfully applied by Fava and co-workers in the assessment of the efficacy of different slurry-phase bioremediation strategies developed for actual-site soils contaminated by PCBs [14,47,71,72,74,75,85] and PAHs [11]. The same tests along with other contact tests based on the use of selected bacteria have been applied in the monitoring of the toxicity of hydrocarbons-contaminated soils subjected to bioremediation under slurry but also solid-phase pilot-scale conditions [73].

## Conclusion

SB is an *ad situ *and *ex situ *technology that can be used for bioremediation of problematic sites (when the less expensive natural attenuation or stimulated *in situ *bioremediation are not feasible), such as those characterized by soils with high contents of clay and organic matter, contaminated with pollutants that are recalcitrant, toxic, and display hysteretic behavior, or when bioremediation should be accomplished in relatively short times under strong pressure and monitoring of environmental agencies and regulators. SB technology allows for the convenient manipulation and control of several environmental parameters that could lead to enhanced and faster treatment of polluted soils: nutrient N, P and organic carbon source (biostimulation), inocula (bioaugmentation), increased availability of pollutants by use of surfactants or solvents, or inducing biosurfactant production inside the SB, etc. An interesting emerging area is the use of SB with simultaneous electron acceptors, which has demonstrated its usefulness for the bioremediation of soils polluted with hydrocarbons. Characterization studies of microbial communities of SB are still in the early stages, in spite of their significance for improving reactor operation and design optimization; so far SB are still modeled as "black boxes".

*Inter alia*, we have identified the following avenues of research for SB in the future: (*i*) application of SB with sequential and simultaneous electron acceptors to soils polluted with contaminants other than hydrocarbons (i.e., pesticides, explosives, etc.), (*ii*) evaluation of the technical feasibility of triphasic SB that use innocuous solvents to help desorbing pollutants strongly attached to soils, and in turn, to enhance their biodegradation, (*iii*) gaining deeper insight of microbial communities present in SB with the intensified application of molecular biology tools such as PCR-DGGE, PCR-TGGE, ARDRA, etc., (*iv*) development of more representative ecotoxicological assays, more complex however more informative than the mere uni-species bioassays or battery of uni-species tests (for instance, microcosm and mesocosms bioassays) to better assess the effectiveness of a given bioremediation process.

## Abbreviations

A: Aerobic; AEF: Availability enhancement factor; CMC: Critical micellar capacity; CDs: Cyclodextrins; 2,4-D: 2,4-dichlorophenoxyacetic acid; 2,4-DNT: 2,4-dinitrotoluene; 2,6-DNT: 2,6-dinitrotoluene; DEP: di-ethyl phthalate; EPTC: S-ethyl dipropylthiocarbamate; ETP: Effluent treatment plant; HCH: Hexachlorocyclohexane; CH: Coefficient of hysteresis; LO: Lubricating oil; PCR: Polymerase chain reaction; PAHs: Polynuclear aromatic hydrocarbons; PCB: Polychlrorinated byphenyls; TPH:Total petroleum hydrocarbons; 2,4,6-TNB: 2,4,6-trinitrobenzene; 2,4,6-TNB 2,4,6-trinitrotoluene; SB: Slurry bioreactor; TLP: Three-phase bioreactor; TPH: Total petroleum hydrocarbons

## Authors' contributions

All authors participated equally in the conception and writing of this review. All authors read and approved the final manuscript.
